# Treatment delay among tuberculosis patients in Tanzania: Data from the FIDELIS Initiative

**DOI:** 10.1186/1471-2458-11-306

**Published:** 2011-05-12

**Authors:** Sven Gudmund Hinderaker, Simon Madland, Martin Ullenes, Donald A Enarson, ID Rusen, Deudatus Kamara

**Affiliations:** 1 Centre for International Health, University of Bergen, Årstadveien 21, N-5009, Bergen, Norway; 2 The International Union against Tuberculosis and Lung Disease, 68 boulevard Saint-Michel, 75006 Paris, France; 3 National TB and Leprosy Programme, Ministry of Health and Social Welfare, P.O.Box 9083, Dar es Salaam, Tanzania

## Abstract

**Background:**

Several FIDELIS projects (Fund for Innovative DOTS Expansion through Local Initiatives to Stop TB) in Tanzania were conducted by the National Tuberculosis and Leprosy Programme (NTLP) during the years 2004-2008 to strengthen diagnostic and treatment services. These projects collected information on *treatment delay *and some of it was available for research purposes. With this database our objective was to assess the duration and determinants of treatment delay among new smear positive pulmonary tuberculosis (TB) patients in FIDELIS projects, and to compare delay according to provider visited prior to diagnosis.

**Methods:**

Treatment delay among new smear positive TB patients was recorded for each patient at treatment initiation and this information was available and fairly complete in 6 out of 57 districts with FIDELIS projects enrolling patients between 2004 and 2007; other districts had discarded their forms at the time of analysis. It was analysed as a cross sectional study.

**Results:**

We included 1161 cases, 10% of all patients recruited in the FIDELIS projects in Tanzania. Median delay was 12 weeks. The median duration of cough, weight loss and haemoptysis was 12, 8 and 3 weeks, respectively. Compared to Hai district Handeni had patients with longer delays and Mbozi had patients with shorter delays. Urban and rural patients reported similar delays. Patients aged 15-24 years and patients of 65 years or older had longer delays. Patients reporting contact with traditional healers before diagnosis had a median delay of 15 weeks compared to 12 weeks among those who did not. Patients with dyspnoea and with diarrhoea had longer delays.

**Conclusion:**

In this patient sample in Tanzania half of the new smear positive pulmonary tuberculosis patients had a treatment delay longer than 12 weeks. Delay was similar in men and women and among urban and rural patients, but longer in the young and older age groups. Patients using traditional healers had a 25% longer median delay.

## Background

In order to better control tuberculosis (TB) and reduce patients' suffering a timely diagnosis and initiation of treatment is crucial. Early treatment reduces the number of micro-organisms released from patients to the environment, it reduces the suffering of the patients and minimizes sequelae in the lungs and body. The potential for finding ways of shortening the duration of the disease and hence reduced transmission gives treatment delay a high priority among topics for study in global TB control, whose central policy is the DOTS strategy (Directly observed treatment - short course).

A recent systematic review showed that some characteristics of patients are associated with delayed treatment initiation [1]. Longer delays were found among patients with pre-existing chronic cough, patients with negative sputum smear, and patients living in rural areas or with geographical barriers. Another systematic review of delay in sub-Saharan Africa showed that travel time and consultation with a traditional healer was associated with delay [2]. A small study from Rwanda did not show longer delays among HIV infected TB patients, but studies are not consistent in this matter. The duration and the importance of the risk factors vary substantially according to geography and culture [3]. In a study in Tanzania the median total treatment delay was estimated 90 days (13 weeks) [4], and another study from same country showed a median patient delay of 120 days (17 weeks) [5]. Initial care-seeking from traditional healers may increase the delay [5,6].

Globally, the WHO targeted to reach 70% detection of new TB cases by 2005 but achieved only around 55% [7]. The Fund for Innovative DOTS Expansion through Local Initiatives to Stop TB (FIDELIS) was created by the Canadian International Development Agency (CIDA) to assist the TB control system to detect "missing" cases through DOTS expansion - and the International Union Against Tuberculosis and Lung Disease (The Union) was selected to manage the initiative [8]. A one-year grant was given to 51 successful applicants in 18 countries where the guiding principle was to support *local *initiatives of *innovative *approaches to detect additional new smear positive cases (NSP), with an emphasis on patients with *limited access to health services*, while maintaining a high cure rate. The results of the various interventions have been analysed [9]. Every patient's access to health services was assessed by determining the duration of symptoms prior to diagnosis and initiation of treatment, in addition to health seeking activities before diagnosis. This created for each project a valuable database on treatment delay of their patients, enrolling all the relevant patients notified in the respective districts. This database was not designed for scientific analysis, but to monitor progress toward targets for project management. The National TB and Leprosy Programme (NTLP) in Tanzania received such a one-year grant in four out of six FIDELIS application rounds of between 2004 and 2008. Our hypothesis was that patients who visit traditional healers have longer delays. Using the existing routinely collected information our objective was to assess the duration and determinants of treatment delay of TB patients enrolled in Tanzanian FIDELIS projects, and to compare the treatment delay by health care provider visited prior to diagnosis.

## Methods

The location of the study was six districts in rural and urban settings of various regions in Tanzania. A total of 57 districts had been involved in the FIDELIS projects of Tanzania between 2004 and 2008, selected by the NTLP based on the district performance and pre-project needs assessment. There are around 130 districts in Tanzania, with approximately 25,000 new smear positive TB patients notified annually. In the six study districts the person responsible for reporting had taken care of all FIDELIS forms, including questions about treatment delay. The forms had been routinely filled, and the forms were collected at the end of the project; unfortunately, all the other districts had discarded their forms by the time of this analysis. In Tanzania TB is diagnosed and treated at most public clinics and selected private clinics, all supported and supervised by the NTLP and reporting their TB cases.

The study had a cross sectional design using data routinely collected from new smear positive TB patients at treatment initiation by the clinician or nurse dealing with these patients. Every new smear positive patient was diagnosed based on detecting acid-fast bacilli in at least two out of three sputum smears, and was interviewed with a short questionnaire about symptom duration from onset until diagnosis, and the health care seeking activities during this time i.e. visiting public clinics, private practitioners, or traditional healers. The information recorded included age, sex, district, symptoms and their duration, and visits to care provider prior to diagnosis. The participants were enrolled between October 2004 and April 2007.

The drugs of all the participants were provided by the National TB and Leprosy Programme (NTLP) through the routine services from either public or private partners reporting to the NTLP. In most instances the person(s) responsible for the TB register at the facilities conducted the short interviews required by the project and sent to the NTLP headquarters the results together with the case finding and treatment outcomes reports. The questionnaires were kept at the districts and these were later collected by NTLP, who gave permission to use the data for research purposes. The data from the forms were double-entered anonymously into the computer by EpiData software and validated, and exported to SPSS for further analysis. Simple frequencies and cross tabulations were undertaken, and median and mean delay was calculated to estimate central tendency. We used 95% confidence intervals as estimates of precision. Non-parametric tests (Mann-Whitney) were used to identify statistically significant differences in delay. Multiple logistic regression was used to quantify the risk factors for patients having a treatment delay of 12 weeks or more, and we included in the regression model the variables district, sex, age group, cough, haemoptysis, fever, and weight loss. Multiple linear regression was used to quantify the excess delay for the risk factors, and because of markedly skewed data on delay we analysed logarithmically transformed delay adjusting by the same variables.

Ethics approval was discussed with the director of the National Institute of Medical Research which is the body responsible for medical research ethics, and we were told by email that as retrospective analysis of routine work it did not require ethical permit in Tanzania. Ethics approval was obtained from the Ethics Advisory Group of The Union, as this is required for any research activity undertaken by consultants in this organization.

## Results

Between 2004 and 2007 the study enrolled 1,161 new smear positive TB patients out of 11,375 patients registered in all FIDELIS projects in Tanzania. The participants had a mean treatment delay of 15.2 weeks (95% CI 14.5-15.8), and the 25-, 50-, and 75-centile was 8,12 and 20 weeks, respectively, demonstrating that the frequency distribution of treatment delay was skewed with a tail to the right.

Table 1 shows the treatment delay of the participants by district, setting, sex and age. The district of Handeni shows a longer delay (median 14 weeks) and Mbozi a shorter delay (median 10 weeks) compared to the other districts (12 weeks). Males and females had similar delay, as did most age groups, except ages 15-24 and older than 65 with longer delay. Urban and rural dwellers had similar delay.

**Table 1:** Treatment delay among participants with new smear positive pulmonary tuberculosis in Tanzania.

	n	Mean Delay (weeks)	95% CI for mean	Median delay (weeks)	Mann-Whitney test
**All**	**1161**	**15.2**		**12**	
					
**District**					
Handeni	75	19.2	(16.5-22.0)	14	p < 0.01
Hai	264	12.1	(11.1-13.1)	12	reference
Lushoto	12	12.6	(12.2-13.0)	12.5	p = 0.8
Mbeya	518	16.9	(15.7-18.1)	12	p < 0.01
Mbozi	243	13.7	(12.5-14.9)	10	p = 0.17
Muheza	49	15.6	(14.1-17.1)	12	p < 0.01
					
Setting					
Urban	345	15.9	(14.5-17.2)	12	p = 1.0
Rural	816	14.9	(14.1-15-6)	12	
					
**Sex**					
Males	680	14.4	(13.6-15.2)	12	
Females	476	16.3	(15.2-17.5)	12	
Missing	5	11.6		12	
					
**Agegroup**					
0-4y	-	-		-	
5-14y	24	18.8	(13.9-23.6)	14.5	p = 0.04
15-24y	186	15.3	(13.7-17.0)	12	p = 0.69
25-34y	430	15.1	(13.9-16.2)	12	reference
35-44y	303	15.4	(14.1-16.8)	12	p = 0.99
45-54y	122	14.1	(12.1-16.1)	12	p = 0.36
55-64y	50	14.3	(11.7-16.9)	12	p = 0.83
65+y	35	17.8	(13.8-21.8)	14	p = 0.03
Missing	11	10.55		12	

Table 2 shows symptom duration and treatment delay according to the symptoms experienced by the patients. Patients who indicated dyspnoea as a symptom had a longer median delay than those who did not, as did patients who indicated diarrhoea. The median duration of cough was 12 weeks, whereas for weight loss it was 8 weeks, and for haemoptysis it was 3 weeks.

**Table 2:** Symptom duration and treatment delay of TB patients in the study.

	Symptom duration (weeks)			Treatment delay (weeks)		
		
Symptom	n	Mean	Median	n	Mean	Median
**All**				1161	15.2	12
Missing				18		
						
Cough						
No	2			2		
Yes	1174	13.5	12	1158	15.2	12
Missing	3			19		
						
Hemoptysis						
No	921			921		
Yes	182	5.6	3	182	16.1	12
Missing	76			76		
						
Fever						
No	1					
Yes	1053	10.0	8	1080	15.3	12
Missing	125					
						
Weight loss						
No	14					
Yes	978	10.2	8	1025	15.7	12
Missing	187					
						
Other complaints						
Chest pain				661	14.5	12
Dyspnoea*				79	19.3	15
Weakness				237	15.3	12
Anorexia				57	15.9	12
Diarrhoea*				16	22.5	20

*Mann-Whitney test p < 0.05, comparing delay in cases who reported symptom with those who did not.

Table 3 shows treatment delay according to health services consulted before diagnosis of their current disease. Patients who had been to a traditional healer had a median treatment delay of 15 weeks compared to 12 weeks among those who had not (p < 0.001); in urban settings 20.8% reported such a visit and in rural areas 19.4%. The patients who had consulted a traditional healer visited him a median of 5 weeks before diagnosis. Patients who had consulted a private practitioner had slightly shorter delay than those who had not.

**Table 3:** Health services consulted before diagnosis, and corresponding treatment delay in FIDELIS projects in Tanzania.

			Delay (weeks)		Mann-Whitney
	n	(%)	Mean	Median	test
**Visited traditional healer before**	924	100%			p < 0.01
-yes	183	20%	18.6	15	
-no	741	80%	14.9	12	
**Visited private clinic before**	962	100%			p = 0.19
-yes	431	45%	14.7	12	
-no	531	55%	16.3	12	
**Visited public facility before**	1024	100%			p = 0.01
-yes	665	65%	16.4	12	
-no	359	35%	13.8	12	

Multiple logistic regression analysis showed that patients who had experienced weight loss had more than 3 times higher adjusted odds ratio (OR 3.4; 95% CI 2.3-5.1) of having treatment delay for 12 weeks or more, and patients with haemoptysis had a 60% higher odds ratio (OR 1.6; 95% CI 1.1-2.2). In multiple linear regression of logarithmically transformed delay we found that patients with weight loss had 1.5 weeks longer delay (95% CI 1.3 - 1.7) than patients not reporting weight loss.

## Discussion

Delay in care seeking is a major challenge for patients with TB and is made worse for the poor [10], contributing to an ever increasing cycle of poverty. Consequently, understanding and addressing this problem is crucial to improving health and reducing poverty. Other studies have highlighted the role of traditional healers in contributing to delay in care seeking for TB in rural Tanzania [11] and in other low-income countries [12-15]. Our study has shown that 50% of the new smear positive patients in selected districts of Tanzania suffer from symptoms for 12 weeks or more before they are diagnosed with TB and treatment is initiated. The mean delay was 15.2 weeks, showing a skewed frequency distribution. Our data covers around 10% of TB patients in the FIDELIS projects in the country and the numbers equal around 4% of annual new smear positive cases in Tanzania [7]. Project reports from two of these projects with over 6000 patients showed that 36% of the patients indicated their treatment delay was 12 weeks or more. In our study a higher proportion was delayed, so these findings may not be representative for the whole country. Tanzania has more than 100 ethnic groups with different cultures, but the urbanization during the last decades may have created a more uniform culture and some loss of ties to extended family and ethnic group. Health seeking behaviour is markedly influenced by location and culture, as shown in the review mentioned earlier [3].

Before receiving their diagnosis 40% of the participants had seen a private practitioner, and 20% of respondents reported consulting a traditional healer, with similar proportions for urban and rural dwellers. In a study in Mwanza region the proportion if TB patients visiting traditional healer was 38.9% in 1998 [5], and a study from Pwani region in 2007 showed that 7.1% had consulted traditional healer [4]. Half of our patients had seen the traditional healer 5 weeks or less before the time of their diagnosis. There is a potential for earlier detection of TB in these patients if the traditional healers would refer all coughing patients to a diagnostic clinic. However, this would require good communication with this sector, as has been demonstrated in other settings such as Malawi [16]. The data regarding traditional healers may be biased utilizing the interview format, as patients may be reluctant to divulge this care-seeking behaviour to professional health care providers. We have no information of the magnitude of this potential information bias.

Patients in Handeni district had longer median delay than Hai, and Mbozi had shorter delays. This is somewhat expected due to a greater scarcity in diagnostic facilities as indicated by the number of *new *sputum smear fixing sites in the FIDELIS project in addition to the old ones; Mbozi got two extra sites and Handeni got five extra sites based on a pre-project needs assessment. Even though Mbozi district has 30% more people, the project planned only two extra smear fixing sites. Urban and rural dwellers of Mbeya had similar duration of delay, and also similar utilization of traditional healer.

We note that the reported duration of cough is basically the same as the total duration of the treatment delay, and underscores that cough is often the first and most consistent symptom in pulmonary TB. Weight loss was a very common symptom but its duration was shorter than the total duration of delay, only 8 weeks. This is expected because weight loss takes some time to appear visibly in the body and would be expected a bit later than the cough. In line with this, haemoptysis had a duration of only three weeks, and may in these cases have been the symptom that precipitated the patient to seek help.

Patients with dyspnoea had a longer delay, which may indicate that their disease was more advanced. Also, patients who mentioned diarrhoea as a symptom had a longer delay. We have no data to speculate on the causes of this, e.g. HIV status. Weight loss was a clear risk factor for a prolonged delay, and this makes sense as weight loss takes some time to manifest itself. In our skewed data the odds ratio of this variable cannot not be taken as an approximation of risk (see later). However, linear regression with log-transformed delay showed that patients reporting weight loss have around one and a half week longer delay than patients not reporting weight loss.

Our study has a number of potential weaknesses. It was not population based, but facility-based covering all government facilities in the selected districts, and a selection of private clinics routinely reporting to NTLP. However, in Tanzania the large majority of TB patients are diagnosed at facilities reporting to the NTLP. Also, our data was comprised of a selection of interview forms that were managed and stored by the district TB coordinator (DTLC). This could indicate a self-selection of districts where the DTLC is more conscientious and provide better services, even though there is no evidence for this potential bias. Furthermore, information bias may have arisen during the interviews depending on the beliefs of the interviewer. We have no information on this, and no quality assurance measures were done for the interview, nor for quality of the forms. These biases may give wrong estimates of delay, and we do not know whether this is consistent in the various locations. There were also some methodological weaknesses. In logistic regression analysis the resulting *odds ratio *is representative for the *risk ratio *if the outcome is fairly rare. Hence, the exact odds ratios of *common *outcomes should not be interpreted as valid *quantification *of risks. Regression modelling is usually not advised for skewed data, unless the dataset is large. We therefore compared with log-transformed data on delay.

## Conclusion

In our study half of the new smear positive pulmonary patients reviewed had a delay of 12 weeks or more, and visiting a traditional healer prolonged the delay. Rural and urban patients reported similar delay. Patients aged 15-24 years and 65 years and older had longer delay than patients aged 25-34 years. Delays in patients with symptoms of dyspnoea and weight loss may simply indicate longer duration of disease.

## Abbreviations

DOTS: Current TB control strategy; DTLC: District TB and Leprosy coordinator; FIDELIS: Funds for DOTS expansion through local initiatives to expand DOTS; NTLP: National TB and Leprosy Programme in Tanzania; TB: tuberculosis; The Union: The International Union against Tuberculosis and Lung Disease.

## Competing interests

The authors declare that they have no competing interests. Several authors were working with the FIDELIS initiative: DK was a project manager of FIDELIS in Tanzania, SGH was an external monitor of FIDELIS in Tanzania, DE and IDR were international directors of the FIDELIS initiative.

## Authors' contributions

SGH developed the protocol, analysed and interpreted the results, and wrote the first draft. SM and MU performed data entry and reviewed the drafts. DK was field project manager and collected the filled forms and reviewed the drafts. DE and IDR discussed the results and reviewed the drafts. All authors accepted the final manuscript.

## Pre-publication history

The pre-publication history for this paper can be accessed here:

http://www.biomedcentral.com/1471-2458/11/306/prepub
